# Nurses’ Perceptions and Expectations of Patient Violence: Language Matters

**DOI:** 10.3390/nursrep15030085

**Published:** 2025-03-01

**Authors:** Darcy Copeland, Mary Potter, Susan Tipton, Debra Culter

**Affiliations:** 1 St. Anthony Hospital, CommonSpirit Health Mountain Region, Lakewood, CO 80228, USA; 2 School of Nursing, University of Northern Colorado, Greeley, CO 80639, USA; 3 Advent Health Parker Adventist Hospital, Parker, CO 80138, USA; 4 CommonSpirit Health Southern Colorado, Mercy Hospital; Durango, CO 81301, USA; 5 Advent Health Avista Adventist Hospital, Louisville, CO 80027, USA

**Keywords:** workplace violence, patient violence, zero tolerance, hospitals, nursing

## Abstract

**Background**: Patient violence is a serious occupational risk for nurses. Some professional rhetoric presents this risk as not part of nursing work, discounting widespread exposure. There is a disjunction between nurses’ experiences and the discourse they are exposed to. There is little to no evidence indicating whether nurses think it is possible to eliminate patient violence or whether their expectations of exposure to patient violence align with the significant risk they face. **Purpose**: The purpose of this analysis was to examine nurses’ perceptions related to the elimination of, expectations of, and desired state regarding exposure to patient violence. **Methods**: This study used a cross-sectional, descriptive design. **Results**: Nearly 500 nurses from seven acute care hospitals in the western United States responded to the electronic survey. Most nurses (85%) do not think it is possible to prevent violence in acute care facilities. Most (81%) also agree that nurses in acute care facilities *do* expect to be exposed to patient violence while at work. A smaller majority (68%) responded that nurses *should* expect to be exposed to patient violence while at work. Respondents indicated that expecting patient violence was an important mechanism in preventing and responding to it. Nurses did not conflate expectations of patient violence with acceptance of patient violence. **Conclusions**: Expecting patient violence is not equivalent to accepting violence, yet these two ideas are often used interchangeably in workplace violence initiatives. Eliminating patient violence is not entirely possible given known risk factors. Enforcing ‘zero tolerance’ to patient violence is untenable, and the inability to enact it may result in frustration among nurses. Language matters, and what nurses are experiencing must be differentiated from an ideal state. The professional narrative about patient violence may have negative consequences and is not aligned with nurses’ experiences or expectations.

## 1. Introduction

Exposure to violence is a well-established occupational risk for nurses. According to the United States’ Bureau of Labor Statistics, the distribution of nonfatal occupational injuries associated with violence is 4.2% for all occupations and 12.2% for nurses; nurses are physically injured due to workplace violence at a rate three times higher than other full-time workers [1]. Fortunately, most incidents of violence against nurses do not result in physical injury. Nevertheless, according to Press Ganey’s most recent safety in healthcare report, there was a 5% increase in reported assaults against nurses in 2023 compared to 2022 [2]. Additionally, nurses practicing in all areas of healthcare may experience assaults, but psychiatric units, emergency departments, adult units, pediatric units, and perioperative units reported more incidents than other hospital units [2]. Finally, perpetrator data is not always reported, but studies have established that patients account for most violence against nurses [3,4].

Based on the available data, it is evident that nurses in hospital settings are exposed to patient violence at disturbingly high rates and frequencies. At the same time, there are workplace violence prevention campaigns purporting that violence “is NOT part of the job!” [5]. There exists a potential contradiction between the lived experience of many nurses and the language in some campaigns addressing violence against nurses; it seems contradictory to state that violence is not part of nursing when one quarter of nurses report having been assaulted while at work [5]. It is worthwhile to pause and reflect on how a nurse recently assaulted by a patient may internalize or respond to being told violence is not part of their job.

There are also workplace violence prevention campaigns that utilize slightly different language to indicate that violence “should not be part of the job” [6]. The shift in language from “is not” to “should not” is important. The former identifies a descriptive state, intended to reflect reality. The latter refers to a desired or ideal state. It is important to accurately distinguish what nurses are experiencing from what nurses ought to experience with respect to occupational risk, in the present case, patient violence. Utilizing clear and accurate language is not a matter of splitting hairs. Different words carry different meanings and evoke different responses.

Another potential contradiction exists in workplace violence campaigns that promote ‘zero tolerance’ approaches. These approaches suggest that people who exhibit violent behavior, including patients, will be withdrawn, removed, or punished in some way [7]. ‘Zero tolerance’ as a policy approach has roots in the criminal justice system [8] and implies that people are responsible for their behavior and must be held accountable [9]. ‘Zero tolerance’ language used in workplace violence campaigns gives the impression that it is permissible and possible to simply remove patients who exhibit violent behavior from hospitals despite established fiduciary relationships, ethical frameworks for safe discharge, and federal laws protecting vulnerable patients. Whether this is the intended purpose of such approaches, this is how they have been interpreted by staff members, leading to a disjunction between policy intent and practical application [7].

It is important to pay attention to disciplinary and institutional language related to patient violence because this is one of the most serious occupational hazards impacting nurses. Nurses’ experiences need to be accurately reflected in the messages that are used. Available statistics indicate that patient violence is, in fact, a part of many nurses’ jobs; whether it ought to be is a different issue. This rhetorical differentiation between descriptive (what is) and normative (what ought to be) states has implications and has not been investigated among nurses. Therefore, a preliminary exploration of hospital nurses’ perceptions related to exposure to patient violence, expectations of exposure, and alignment between these and their desired state was undertaken.

The purpose of this analysis was to examine nurses’ perceptions related to the elimination of, expectations of, and desired state regarding exposure to patient violence. The data came from a larger parent study examining factors associated with nurses’ decisions to restrain and/or seek legal action when patients exhibit violent behavior. Participants were asked the questions presented here. They were then presented with three unfolding case studies and were asked at what point in each they would restrain and/or seek legal action against the hypothetical patient. Results from the analyses of the case study responses are not presented here.

## 2. Materials and Methods

### 2.1. Design

This study used a cross-sectional, descriptive design.

### 2.2. Recruitment

All registered nurses from seven hospitals within a healthcare system in the western United States, six suburban and one rural, were eligible to participate. Upon receipt of IRB approval, the survey invitations were sent via organizational email. Following the initial invitation to participate, reminder invitations were sent once per week for four weeks. Consent to participate in the research study was implied if participants continued to the survey hyperlink after reading the recruitment email outlining the study’s purpose, procedures, risks/benefits, their ability to skip questions or exit at any time, and who to contact with concerns about the study. A sample size of 385 was estimated to be needed to be representative based on an unknown population of nurses employed at the seven participating hospitals and using a confidence interval of 95%, a population proportion of 50%, and a margin of error of 5%.

### 2.3. Data Collection

What is reported here are only results from three yes/no questions included in the investigator designed survey: do you think it is possible to prevent patient violence in acute care facilities (hospitals) and get to zero instances of patient violence, do nurses in acute care facilities (hospitals) expect to be exposed to patient violence while at work, and should nurses in acute care facilities (hospitals) expect to be exposed to patient violence while at work? Each of these questions was followed with a free text box, and participants were asked to explain why or why not. In the parent study, the yes/no responses to these questions were examined in relationship to when and if participants indicated they would restrain and/or seek legal action against the hypothetical patients presented in the three unfolding case studies. Presented here is an analysis of the written explanations participants provided in response to each of these questions.

### 2.4. Data Analysis

Descriptive statistics were used to summarize data. Chi square tests were used to examine relationships between categorical variables. Fisher exact tests were used to examine associations between study variables when cell frequencies were less than five. Odds ratios were used to further examine the strength of the relationship between variables.

The free text responses for each of the three questions were separated by whether the respondent indicated yes or no to the question. Analysis of responses occurred question by question, one at a time. The aim of the analysis was to identify patterns within the responses. Braun and Clark’s [10] steps for thematic analysis were followed to provide a systematic process: familiarization with the data, generation of initial codes, searching for themes, reviewing themes, defining and naming themes, and writing. Textual responses to each question were read in their entirety and coded individually by all members of the research team. Each team member analyzed their own codes and organized them into overarching themes independently. Team members then came together to discuss and refine themes for each question’s yes and no responses. Notes were taken at each of these meetings and serve as an audit trail for the group’s decision-making; each team member was responsible for keeping their own notes related to individual coding and theming. Once themes were agreed upon, team members individually wrote descriptions and identified supporting quotes from the responses and then came together to develop a collaborative description and choose respondent quotes that best illustrated the identified theme. Respondent quotes are presented exactly as they were written. Multiple team meetings occurred over a period of seven months to discuss each of the questions. These meetings were an important mechanism to reduce the potential for individual bias in reporting.

## 3. Results

### 3.1. Sample

The response rate to the survey was 499/3780 (13%). Incomplete survey data was retained for analysis; therefore, reported individual question response rates may differ. Most respondents were female (277, 75%), 27 (7%) were male, 1 (0.3%) was non-binary/non-conforming, and 64 (17%) did not respond. Most respondents had between 1 and 10 years of experience as an RN (156, 42%), followed by those with 11 or more years of experience (138, 38%), and only 11 (3%) had less than one year of experience; 64 (17%) did not respond. Most respondents reported they were white (249, 68%), followed by Native American Alaskan Native (11, 3%), multiracial (8, 2%), Asian Pacific Islander (7, 2%), Middle Eastern North African (2, 0.5%), and black (2, 0.5%). A total of 19 respondents (5%) reported they were Hispanic/Latino, and 71 (19%) did not report any race or ethnicity data. Sample age, education, department, and role demographics are presented in Table 1.

### 3.2. Prevention and Expectation

Most respondents reported that they did not think it was possible to move to zero instances of patient violence, that nurses do expect to be exposed to patient violence, and nurses should not expect to be exposed to patient violence (see Table 2). Themes from the written responses to each of these questions, based on affirmative or negative responses, can be found in Table 3, Table 4 and Table 5.

Race was significantly associated with a perception that it was possible to prevent patient violence in hospitals *X*^2^ (6, *N* = 302) = 19.95, *p* = 0.003. Unit worked *X*^2^ (18, *N* = 303) = 36.02, *p* = 0.007, and education *X*^2^ (3, *N* = 302) = 9.24, *p* = 0.026, were significantly associated with whether nurses do expect to be exposed to patient violence. More males than females reported that nurses should expect to be exposed to patient violence *X*^2^ (2, *N* = 305) = 7.23, *p* = 0.026.

There was a significant association between the perception that it was possible to prevent patient violence and whether RNs do expect to be exposed to patient violence *X*^2^ (1, *N* = 369) = 5.66, *p* = 0.017, and whether RNs should expect to be exposed to patient violence Fisher’s exact 2-sided (*p* < 0.001). The odds of reporting it was possible to prevent patient violence were significantly lower among those who agreed that nurses do expect to be exposed to patient violence (OR = 0.464, 95% CI [0.244, 0.883]) and among those who agreed that nurses should expect to be exposed to patient violence (OR = 0.107, 95% CI [0.033, 0.351]).

***Is it Possible to Prevent Patient Violence in Hospitals and get to Zero Instances?***

Three hundred and eighty-one nurses wrote a response to this question. A sentiment that crossed both the yes and no explanations was lack of mental health resources. A mental health crisis was identified as a major contributor to the expression of violent patient behavior. Needing “better mental health treatment and resources” was seen as both a way to prevent violence and as a reason it is currently not possible to prevent violence.

Three additional themes were identified in the written responses from those who felt it was possible to achieve zero instances of patient violence in acute care settings and in the narratives from those who thought it was not possible to eliminate patient violence in acute care settings (see Table 3).

**Yes**

**Leadership Support.** It was felt that leaders needed to prioritize staff, not patient, safety. The needs of staff were pitted against patient satisfaction ratings, for example, “Actions should have consequences and management should worry more about ensuring the safety of their staff than they do about satisfaction surveys”. Two subthemes, staffing and security presence, were identified.

***Staffing.*** Support from leaders to provide adequate staffing was described as particularly useful in achieving zero instances of patient violence: “I think the setting can be controlled enough (i.e., low nurse to patient ratios, enough ancillary staff) to seriously prevent instances of patient violence”.

***Security.*** In addition to the adequacy of nursing and ancillary care staff, increased security presence was also identified: “It can be done but requires stronger security forces and employees feeling supported and safe by their organizations to limit behavior”.

**Staff Training and Behavior.** Staff training, including de-escalation and situational awareness, was identified as a way to achieve zero violence: “I think we don’t do enough to de-escalate situations. I also think we need more education on keeping ourselves safe and identifying potential threats”. Staff behaviors such as setting clear expectations, walking away from situations, and being mindful of their own role in escalating situations were also raised: “The way we react to patients can reduce a lot of violence; nurses are not well educated on how to deal with psychiatric patients”. A specific element of training and behavior that was identified was early intervention.

***Early Intervention.*** Providers’ prompt response to warning signs of escalating behavior was identified as another way to reach zero instances of patient violence: “Some doctors are very lenient and not so good about medicating these patients before they become violent”. Early intervention was tied to staff situational awareness: “Patients most of the time become violent and aggressive when their needs are not met (physical, psychological, spiritual). These can be addressed at an earlier stage to prevent violence”.

**Patients Controlling Their Behavior.** While some respondents acknowledged the role of altered mental status in patient violence, several felt the onus was on patients to control their behavior. One person wrote, “People always can choose to make the right choices”, and another, “I believe it is possible, yes. Unfortunately, it’s up to the patients to be decent human beings which we can’t control”.

***Accountability for Behavior.*** In addition to patients controlling their own behavior, respondents felt that accountability would eliminate patient violence. One respondent wrote, “I think there would be no violence against healthcare workers if there were consequences that the public knew about. Assault is assault no matter what the profession and patients who are violent need to be held accountable”. Another said, “People should be immediately given behavior contracts. They should not be coddled. Any instances of threats or violence should be reported to police”.

**No**

**Patients are Human, and Sick, and Hospitalized.** Many respondents commented that patients are human beings, and “People are unpredictable. We never know when violence will be exhibited or how. This is an unfortunate truth in the healthcare field”. This unpredictability creates an ever-present risk: “We can never predict with certainty a person’s reaction. I believe there will always be some margin of risk when working with humans”. Nurses’ ability to control those unpredictable behaviors was also brought up: “We cannot control people, not their emotional/psychological states nor their triggers”.

Many respondents attributed the inevitability of patient violence to altered mental status, arising from a variety of sources: dementia, psychosis, delirium, encephalopathy, traumatic brain injury, stroke, tumors, substance intoxication and withdrawal. Human unpredictability, altered mental status, and additional factors such as fear, stress, and pain intersected: “Disease processes such as delirium brought on by UTI in older patients or agitation in alcohol withdrawal patients; untreated mental health issues in any patient; fear/anxiety/stress related to the hospital stay. You will never be able to get rid of this so therefore potential for patient violence will always exist”.

**Patients Choose To Be Violent**. Many respondents felt it would not be possible to eliminate patient violence because violence exists in society at large: “There are many violent people in the world, and they get sick and need medical care just like everyone else”. People behaved in the hospital the same way they behaved out of the hospital: “Some people are just mean and think they can be violent, sexually inappropriate, verbally inappropriate towards medical staff”. Patients were viewed as exercising free will: “Patients always have the free will to choose violence as a means of communication”. Choosing to exhibit violent behavior was also attributed to patient entitlement: “People feel entitled to do whatever they want”.

**Can’t Prevent It But We Can Mitigate It**. The final theme identified among responses of those who felt it was not possible to eliminate patient violence in hospitals was the idea that while it cannot be eliminated, more can be achieved to mitigate it. Mitigation was tied to patient unpredictability: “We can improve our prevention strategies, but ultimately we cannot control our patients’ behavior”, and “Although ultimately I think harm to staff can be mitigated it is unrealistic to expect patients to never react in a violent way”. Additionally, mitigation was tied to training and related to both the frequency and severity of violent occurrences: “I believe there will always be situations that cause people to act out in a verbal or physical manner. I do believe training staff to mitigate these situations could decrease the severity and/or minimize the incidences”.

***Do* Nurses in Hospitals Expect to be Exposed to Patient Violence While at Work?**

Three hundred thirty-eight nurses wrote a response to this question. Five themes were identified in the written responses from those who responded affirmatively and four themes were identified in the responses from those who responded negatively (see Table 4).

**Yes**

**Normal Part of the Job**. Many participants framed patient violence as an inherent part of nurses’ work, using phrases like “a hazard of the trade”, “comes with the territory”, and describing it as something nurses knew was “part of the job”. It was also described as timeless: “Nurses have always been victims of violence”. Although patient violence is not novel, it was perceived as occurring more often than in the past: “It is something that has always happened, it is currently escalating in frequency”. The frequency of exposure to patient violence, “all the time” is what rendered it both normal and a part of their jobs: “It’s become a normal expectation unfortunately just because it happens so much”. In addition to being a frequent occurrence, it was also described as a universal experience for nurses: “Every single nurse I have talked to has experienced violence at the bedside”.

**Nature of the Work.** A cascade of circumstances making violent patient behavior likely was uncovered. Participants again described patients as “emotional and will never be entirely predictable”. They are then hospitalized with medical conditions and “life changing situations”. Altered mental status was repeatedly cited as a reason to expect violent patient behavior: “Sick and confused people can be violent when they don’t know who we are or where they are”.

When people who are emotional, unpredictable, sick, and altered are hospitalized, their sense of vulnerability, fear, stress, and lack of control increases and impairs coping. Respondents were aware that “Tensions are often high and with that the risk of violence increases”, that “We deal with vulnerable patients that do not always cope or deal with anger/frustration in a healthy way”, and “Patients feel helpless and tend to act out to feel in control”. Then, “Add on factors like long wait times, lack of resources and staffing issues and it’s likely to happen”. Recognizing this web of circumstances, one participant wrote, “If you deal with any type of patient, it is a risk”.

**Rise of Mental Health Issues and Lack of Mental Health Resources**. The frequency of exposure to patient violence coincided “with a rise in mental health issues”. The increased need for mental health services has not been met with an increased supply of services and “mental health facilities often do not have beds”. The healthcare system was unequipped, or unwilling, to address these needs: “Until the US as a whole changes its approach to how we deal with mental health patients the violence from these patients will continue to exist”. Hospitals that are unprepared to address mental health issues exacerbated the issue: “Psych patients when they are in the height of a crisis are dangerous, and what do we offer them? We give them a cold room with no windows in an uncomfortable gurney surrounded by strangers telling them to ‘calm down’…. Placing an unruly patient in a small room with little to no entertainment until a mental health evaluator can come evaluate them simply to let them know that there is no space available at any facilities only creates a higher level of anxiety and discontent”.

**Customer Service Culture.** Many participants referred to culture as a reason they expected to be exposed to patient violence: “This industry is too focused on customer service and face saving and not enough on the care and safety of the nurses, pushing us to unsafe staffing ratios and allowing a ‘customer is right’ perspective”.

Customer satisfaction was pitted against staff safety: “Because the patient is always top priority and nurses are tertiary”. The reason for the prioritization of customer service over staff safety was that “HCAHPS scores are such a high priority staff has no choice but to be at the beck and call of our patients and sometimes they are very demanding. With that, some get verbally and physically abusive with demands”. Participants also felt blamed for patients becoming violent, “Because the hospital is more concerned protecting themselves than their staff. When attacked by patients we are asked what we could have done differently and how it is our fault”.

**Expectation Not Acceptance.** Participants differentiated expectation from acceptance: “I think they expect it now but don’t accept it”. Statements highlighting a discrepancy between what nurses actually experience at work and what they felt they ought to experience were plentiful: “This does not mean that they SHOULD expect to be exposed to patient violence, but it is common enough that we prepare ourselves for it”, “Because it has been shown time and time again. Doesn’t mean it’s right”, “While I don’t think it’s fair, I do believe nurses expect to be exposed to patient violence”, and “It comes with the territory, even though it shouldn’t”.

**No**

**Expect the Possibility.** Many nurses who responded that nurses in hospitals do not expect to be exposed to patient violence qualified their responses with statements like, “I don’t think any of us expect it. We know it could happen though”, and “I know it happens, but I don’t expect that it will happen”. Participants indicated that while they did not expect to be exposed, they did in fact expect that the possibility existed that a patient could become violent; “I don’t think it is expected to happen to everyone, but I do think it is expected for it to be possible”. The possibility of patient violence was expected, not the reality.

**Don’t Anticipate It.** In contradiction to expecting the possibility of violence, one reason participants gave for not expecting to be exposed to patient violence was that “it just happens” and was therefore not anticipated. Some participants felt patient violence “is always a surprise”. The lack of anticipation created a lack of expectation: “No, this is not something we anticipate happening”.

Some comments related to lack of anticipation were specifically directed to a lack of preparedness in new nurses: “I think this is a big shock for new nurses”. There was a perception that nursing schools did not provide information regarding patient violence or de-escalation training: “Nursing schools do not prepare nurses to defend themselves”.

**Want to be Safe at Work.** One reason respondents gave for not expecting to be exposed to patient violence was that “No one should come to work expecting to be exposed to violence”. Places of employment are expected to be safe for employees, and hospitals are places of employment. One nurse comes “to my job with the expectation that I am going to be safe at work”. Some of these respondents compared employment in a hospital to work in other settings: “You wouldn’t expect to be exposed to violence at any other job, and to me, as a nurse, it’s not expected either”. There was a universal expectation of employee safety regardless of setting.

**Here to Provide Care.** The final theme identified from those who responded that nurses do not expect to be exposed to patient violence was related to nurses’ role as caregiver: “We are here to help patients and families”. Nurses “go to work with the intent of caring for people” and there was an underlying assumption that the intent to help would prevent patients from exhibiting violent behavior; “The hope is that we are there to help and that the ‘good’ in people will outweigh the bad behavior”. Nurses idealized their relationship with patients: “I think we as nurses have this ideal that we are helping people and in turn people may be ungracious but not violent”.

***Should* Nurses in Hospitals Expect to be Exposed to Patient Violence While at Work?**

The first two questions were descriptive in nature, asking nurses to reflect on the current state of their work. This third question was normative, asking nurses to reflect not on what is, but on what ought to be. Three hundred and ten nurses wrote a response to this question.

Four themes were identified in the written responses from those who responded affirmatively, and three themes were identified in the responses from those who responded negatively (see Table 5).

**Yes**

**Because They Are Patients**. The unpredictable nature of humans was again recognized: “Because they are caring for human beings and humans are not perfect”. It was further acknowledged that both illness and environment play a role in patient violence: “Because this is a very stressful environment for a patient for multiple reasons. Not only are you sick, but there’s other factors like unfamiliar environment, missed work, maybe childcare concerns, money, etc”. Providing care to people in compromised states was noted as involving issues of autonomy, independence, fear, grief, trauma, and pain, which contributed to patient violence. It was also highlighted that certain conditions, which predispose people to needing hospitalization are also conditions associated with the potential for violent patient behavior.

***Altered Mental Status and Mental Health Needs.*** Exposure to patient violence was described as “part of working with confused patients”. Caring for patients with conditions that result in alterations in cognition or mental status were cited as reasons nurses should expect to be exposed to patient violence: “If you are working with these types of patients, you should expect the potential for violence”.

**It is Reality.** One reason respondents felt nurses should expect to be exposed to patient violence was that “it is real, it happens”. Because “it is the reality” nurses felt “it is an unrealistic expectation to think that you won’t get hit”. Exposure to patient violence was perceived as an inherent part of their job, and since “it is going to happen. Nurses should be prepared to deal with situations”.

***Need To Be Prepared.*** Nurses should expect patient violence because expectation led to preparation: “Patients can be violent, and everyone should be prepared”. Mitigating potential damage was another important part of preparation, “By being prepared for potentially violent patients, there is an awareness and a plan that can be followed to prevent injuries”. Nurse should expect patient violence because doing so provided a layer of protection and optimized patient care: “If you don’t expect that patients can become violent, you can’t protect yourself and you can’t appropriately care for that patient”.

**Yes, But.** Many respondents qualified their responses. Some qualifications were due to practicality: “from a practical point of view, it’s easier to keep yourself and your coworkers safe if you have an awareness of the possibility of patient violence”. Other qualifications referenced frequency, “but it should not be near as common as it is”. Others compared the current state to an ideal state: “In an ideal world it should not be an expectation, but I would say that yes, it should probably be expected so that at the very least nurses aren’t surprised when it happens”. The primary qualification for yes responses was related to the necessity to anticipate and prepare for it: “Nurses should expect to see violence at work, but it is also essential that they be given training on how to handle aggressive patients”, “I hate that we should anticipate it, but in anticipating it, we learn how to protect ourselves”, and “I don’t want to expect it, but I want to be prepared to handle patient violence if it occurs”.

**Expect ≠ Accept**. The final theme from those agreeing that nurses in hospitals should expect to be exposed to patient violence involved differentiating between expecting it and accepting it: “I think they should expect it, because it is something that is realistic to expect. That doesn’t mean it is a good thing or should be tolerated, but it is a real possibility”. Expectation was a product of reality, but not a desired state: “It SHOULD never happen at work; however, that’s an impossible expectation to have”. Endorsing that one should expect patient violence was not equivalent to saying it was acceptable: “To not have any violence would be grand though it is unrealistic. Not saying it is okay, not saying it is going to happen every day. Just be on the alert”.

**No**

Negative responses to this question were less well articulated than other responses; some responses were not even answers to the question posed. Examples of such responses include “It effects the mental health of caregivers” and “There should be hard limits set for people who infringe on the safety of those caring for them”.

There were also respondents whose rationale for their no response seemed contradictory: “Since nurses deal with people/public and at [their] worst, vulnerable times, it is likely that violence will occur”. Other incongruent responses were related to patient factors previously identified as reasons nurses do expect patient violence, such as “we need to have close contact with our patients” and “Patients may react to meds and become violent or special needs patients react out of fear”.

**No, But.** As with the affirmative responses, many participants who responded negatively qualified their response. “No one should expect to be exposed to violence, but one shouldn’t be surprised when they encounter it at work in a hospital”, and “Nurses should not have to fear for their safety, but it is understood that the risk of violence is ever present”.

***Expect the Possibility.*** One specific qualification was related to training or preparedness, for example, “We should all be prepared for potential violence but never expect it” and “I do not think nurses should expect violence but should be mentally and physically prepared for the possibility of violence”. Nurses should not expect to be exposed to patient violence but should expect that it is a possibility and therefore prepare.

***Contradicts Reality.*** Another specific qualification was that saying no seemed to contradict reality: “They shouldn’t expect to be exposed to violence but unfortunately we are” and “I don’t think anyone should be exposed to violence anywhere, but it is part of being a nurse and working with patients that are possibly cognitively impaired”.

***Compare to Ideal.*** There were also comparisons to an ideal: “In an ideal world and work experience I would say no they should not expect to be exposed to violence at work, but we are not in an ideal world”. Considering work without exposure to patient violence was a theoretical exercise, “In theory, no one should be exposed to violence”, or a dream, “While nice to dream about a job of no violence, it is never going to happen”.

**Employee Status and Workplaces**. There was a general sentiment that people should not be exposed to violence: “No one should ever have to deal with violence” and “Because violence is unacceptable”. This idea extended into the workplace: “No one should expect to have violence happen at work”. Employee rights were also identified as a reason nurses should not expect to be exposed to patient violence: “We have a right to work in an environment where we shouldn’t feel at risk for violence”. The specific location was also implicated, “Because a hospital should be an area of safety and respect”. Comparisons were also made to other jobs and situations.

***Comparison to Other Jobs.*** General comparisons to other workers were made: “Nursing should be as safe as any other career field”, as well as comparisons to high-risk jobs: “No person should be expected to experience violence unless you work in an industry that has a lot of it”. Comparisons were also made to other specific occupations: “Why should anyone at any workplace be expected to put up with and tolerate violence, we don’t expect this of teachers or police officers”, “Lawyers do not go to work expecting to be attacked. Business people do not expect violence”.

***Comparison to Other Situations.*** In addition to comparisons to specific jobs, comparisons were made to other situations: “I would never allow someone in the street to walk up and punch me why should it just be expected at my place of employment”. Respondents compared being exposed to random violence in public to exposure to patient violence while at work: “If a person hit you, spit at you or verbally abused you out on the street or in a store, it is a crime, just because we are nurses does not mean we should tolerate that kind of abuse”.

**Status as a Helper/Caregiver.** A final theme was related to status: “Nurses deserve to be safe while performing work duties”. The discipline’s value of treating all people with respect was reflected back on nurses themselves: “Nurses deserve to be treated with the same respect they treat their patients with”. Nurses’ roles as caregivers was cited, “We shouldn’t be exposed to violence when our job is to take care of people and get them better”. People whose job it is to help people were regarded as having special status: “No one should be expected to experience violence in their place of work, especially those that are trying to help people”.

## 4. Incidental Finding

One incidental finding was not directly related to the purpose of the analysis. It is the number of derogatory and pejorative comments made about patients. It is atypical for nursing literature to highlight such things, but it seems important to do so. Patients were often referred to as “entitled”, “awful”, “jerks”, and “horrible”, and mental health diagnoses were viewed as “excuses” rather than medical issues. Furthermore, beyond accountability, some nurses wanted retribution.

## 5. Discussion

It is difficult to interpret the finding that most hospital nurses do expect to be exposed to patient violence and simultaneously most do not think hospital nurses should expect to be exposed to patient violence. One explanation is simply that they, understandably, do not want to be exposed to patient violence at work and therefore think they should not be. The relationship between participants’ perceptions regarding prevention and expectation is clearer. Those who felt patient violence in hospitals could be eliminated were less likely to expect it, and those who felt it could not be eliminated were more likely to expect it. Participants who do not think it is possible to prevent patient violence identified known risk factors for violence in their responses. A great number of hospitalized patients possess risk factors for violence, and these nurses were therefore more likely to expect to be exposed. If patient violence is interpreted largely as a conscious choice a person is making, it is interpreted as preventable.

These results highlight conflicting interpretations regarding patient violence. Some respondents felt strongly that violent patient behavior was willful, while others interpreted it as a confluence of factors. It is possible that interpretations of events are related to tolerance and/or expectation. For example, patient behaviors in labor and delivery settings are interpreted as expected while the same behaviors are interpreted by medical/surgical nurses as unacceptable [9]. This has important implications. If behavior is interpreted as expected, it might be more tolerated; staff members with more tolerant attitudes have reported less burnout and a greater sense of personal accomplishment [11]. Additionally, while formal determination of behavioral intent is typically beyond the scope of practice for nurses, it is obvious from these responses that it is something nurses often do informally. Ascribing intentionality to patients’ violent behavior has been linked to a heightened emotional reaction and increased likelihood of reporting events [12]. Although responses to patient violence were not directly examined here, there were obvious differences related to intentionality. Interpretation, specifically the concept of intentionality, seems to have an important place in conversations about patient violence.

It is important to note that expecting patient violence was not conflated with accepting patient violence. The frequency of exposure resulted in a degree of normalcy and expectation, but that did not translate to acceptance. Acknowledging that patient violence was expected was not an approval of patient violence. This clear distinction indicates that many nurses are aware of known risk factors for patient violence [13]. It might also indicate a level of tolerance or resilience to patient violence. At the very least, it makes clear that nurses can distinguish between what is occurring around them and what they wish was occurring instead. Professional discourse framing patient violence as something to be expected would align with nurses’ experiences and would not communicate acceptance.

It is understandable that nurses reported wanting to work in safe environments. In the United States, workers have a right to safe work environments, and it is the duty of employers to provide an environment free of known hazards [14]. The comparisons made to other workplaces and other situations, however, are worth examining. Participants drew comparisons to teaching, banking, and law. Teachers, bankers, lawyers, and nurses all enter fiduciary relationships, meaning they are duty bound to act in the best interests of the other party. There are, however, critical differences between nurse–patient relationships and teacher–student, banker–customer, and lawyer–client relationships. Context is a crucial distinction. Hospitalized patients are ill or injured to the point of needing medical and nursing care not available elsewhere. It is an expectation that doctors and nurses utilize their specialized knowledge and skills in service to these vulnerable patients. Nurses in this study did not compare themselves to doctors, who work in the same environment, but to professionals who work in very different circumstances. Similarly, nurses did not compare themselves to professionals working in public safety sectors such as police officers or firefighters. It is not possible to follow up with these responses for clarification; however, the choices of professions used for comparison were noteworthy.

Similarly, nurses’ role as a helper as a rationale for not expecting to be exposed to patient violence is worth exploring. While American nurses are responsible for adhering to the *Code of Ethics for Nurses* [15], there is not a concomitant obligation on the part of patients. The nurse–patient relationship is not quid pro quo. The expectation is that nurses provide care with respect and dignity to all; there is not a contingency statement. This does not, however, diminish the emotion being communicated by respondents. The distress associated with conflict between professional duties and personal feelings in the context of patient violence has been noted [16]. The depth of a nurse’s commitment to patients is perhaps experienced as equally deep betrayal in the context of patient violence.

With respect to the demeaning and derogatory language used, it is understandable that nurses exposed to patient violence want the behavior managed, and they want the opportunity to seek justice if they believe a crime has been committed. These comments might simply indicate the level of frustration nurses experience due to patient violence. At the same time, vengeance is incongruent with the stated values of the profession and likely has a place only in limited, if any, situations. Several of these findings may serve as a call to the discipline to examine professional identity formation and how nursing’s values are internalized. The discipline would benefit from an analysis of what an ethical response to patient violence may look like.

### 5.1. Implications for Practice, Education, and Research

The primary implication for nursing practice and education based on these findings is the need for training related to patient violence. Training that involves enhancing situational awareness has been shown to be effective in decreasing incidents of workplace violence [17]. Other topics include identification of known risk factors for violence, de-escalation techniques, and principles of trauma-informed care. It could also be valuable to remind nurses that all patients have the potential to display violent behaviors; any patient with an altered mental status is to be considered very high risk. Respondents indicated that expecting patient violence led to preparation; anticipating patient violence afforded a level of protection. Training may help to prevent patient violence and result in nurses feeling better prepared.

Another implication for practice is that nurses in acute care facilities do not have adequate mental health resources available for their patients. There is a lack of mental health resources in communities as well as a lack of trained professionals within hospitals. If reducing patient violence is truly a hospital’s aim, it needs to do a better job resourcing mental and behavioral health specialists who can address the needs of patients most at risk of becoming violent. A final practice implication related to these findings is that nurses perceive their leaders as prioritizing patients’ needs over their own needs in the context of patient violence. Ideally, hospitals strive to ensure the health, safety, and well-being of patients and employees. Practice environments should strive to create safe environments, period. Safety is not a zero-sum situation; in truly safe environments, both patients and staff are the beneficiaries, and the adversarial sentiment is eliminated.

There is always a need for research aimed at identifying best practices with respect to prevention, mitigation, and recovery from patient violence. Additionally, the number of nurses who viewed the nurse–patient relationship as a quid pro quo and who used derogatory descriptors of patients was surprising. Investigating the current state of nurses’ understanding of the discipline’s social contract and their professional identity might shed light on this. A final recommendation is to directly test the impact of different messaging and policies on the frequency of patient violence as well as the distress experienced by impacted nurses. Language plays an important role as a potential contributor to violence as well as a mechanism for interpreting and consequently responding to it. These relationships are worth exploring.

### 5.2. Limitations

Due to administrative requests, the survey was distributed between two major U.S. holidays, which may have impacted the response rate. The complete survey was lengthy, requiring approximately 15 min to complete, and some respondents quit prior to completing it. Additionally, several nurses (17%) who completed the survey did not answer all demographic questions, limiting our ability to fully describe the sample. This study did not involve interviews, and due to the anonymous nature of data collection, there was no way to follow up or clarify participant responses.

## 6. Conclusions

This analysis indicates that hospital-based nurses do not think it is possible to eliminate patient violence, expect to be exposed to it, and unsurprisingly, they do not want to be. Disciplinary messaging that violence ‘is not part of the job’ might need re-evaluation. Similarly, it is worth asking, if we cannot achieve zero instances of patient violence, should ‘zero tolerance’ be the aim? There is literature suggesting that the conflict between duty of care responsibilities and zero tolerance policies causes increased stress [18]. Given the amount of distress that is associated with exposure to patient violence and the protective effects of developing tolerance and resilience, perhaps these qualities should be widely endorsed and promoted. Less time and attention can be devoted to promoting ‘zero tolerance’, and more time and energy can be devoted to resilience and trauma-informed care principles [18]. Importantly, as recognized by participants, re-evaluation of the professional messaging related to patient violence is not equivalent to endorsing problematic behavior.

Patient violence is a complex phenomenon with no simple solutions. Nurses’ responses to patient violence are equally complicated; however, overwhelmingly, staff feel unsupported by their organizations and leaders with respect to workplace violence [19]. Contradictory messaging and untenable policy solutions may contribute to this perception. Moving away from framing patient violence as never ok to conceptualizing it as a significant risk associated with working closely with people in distress is not equivalent to saying there is nothing one can achieve to resolve it or that it is acceptable. On the contrary, framing patient violence as one of many occupational risks hospital-based nurses are exposed to may be a powerful shift in not only messaging but also the lived experience of those exposed to patient violence. Framing patient violence as an occupational risk and responding to the distress caused by it as an occupational injury may also help destigmatize patients.

## Figures and Tables

**Table 1:** Sample demographics.

		*n* (%)			*n* (%)
Age Group	20–29	46 (13%)	Department	Wound Care	1 (0.3%)
	30–39	104 (28%)		Quality	1 (0.3%)
	40–49	66 (18%)		Flights	1 (0.3%)
	50–59	48 (13%)		Hospice	1 (0.3%)
	60+	22 (6%)		Case Management	1 (0.3%)
	No Response	83 (23%)		Community	1 (0.3%)
Education	ADN	37 (10%)		Program Coordinator	1 (0.3%)
	BSN	219 (59%)		Neuro ICU	2 (0.5%)
	MSN	43 (12%)		Float Pool	3 (1%)
	Doctoral	3 (0.8%)		Palliative Care	4 (1%)
	No response	67 (18%)		Multiple	7 (2%)
Role	House Supervisor	1 (0.3%)		Step Down/PCU	7 (2%)
	Other	1 (0.3%)		Inpatient Rehab	8 (3%)
	RN Navigator	2 (0.5%)		Interventional	10 (3%)
	Charge Nurse	3 (1%)		Other	13 (4%)
	Prof Dev	3 (1%)		Outpatient Clinic	15 (4%)
	Case Manager	5 (1%)		Labor and Delivery	16 (4%)
	APRN	7 (2%)		Peri-Anesthesia	44 (12%)
	Program Coord	7 (2%)		ICU	52 (14%)
	Management	26 (7%)		ED	53 (14%)
	Staff RN	251 (68%)		Acute Care	72 (19%)
	No response	63 (17%)		No response	66 (17%)

**Table 2:** Nurses’ perceptions of prevention and expectation of patient violence.

	Yes	No
Do you think it is possible to prevent patient violence in acute care facilities and get to zero instances of patient violence?	64 (15%)	367 (85%)
*Do* nurses in acute care facilities expect to be exposed to patient violence while at work?	350 (81%)	82 (19%)
*Should* nurses in acute care facilities expect to be exposed to patient violence while at work?	140 (32%)	292 (68%)

**Table 3:** Themes from affirmative and negative free text responses to question one.

Yes	No
Need better mental health treatment Leadership support Staffing Security Staff training and behavior Early intervention Patients controlling their behavior Accountability for behavior	Need better mental health treatment Patients are human, and sick, and hospitalized Patients choose to be violent Can’t prevent it but we can mitigate it

**Table 4:** Themes from affirmative and negative free text responses to question two.

Yes	No
Normal part of the job Nature of the work Rise of mental health issues and lack of mental health resources Customer service culture Expectation not acceptance	Expect the possibility Want to be safe at work Don’t anticipate it Here to provide care

**Table 5:** Themes from affirmative and negative free text responses to question three.

Yes	No
Because they are patients Altered mental status and mental health needs It is reality Need to be prepared Yes but Expect ≠ accept	No but Expect the possibility Contradicts reality Compare to ideal Employee status and workplace Comparison to other jobs Comparison to other situations Status as a helper/caregiver

## Data Availability

The raw data supporting the conclusions of this article will be made available by the authors upon request.

## Public Involvement Statement

There was no public involvement in any aspect of this research.

## Guidelines and Standards Statement

This manuscript was drafted against the STROBE guidelines for reporting observational studies.
